# A novel statistical method for modeling covariate effects in bisulfite sequencing derived measures of DNA methylation

**DOI:** 10.1111/biom.13307

**Published:** 2020-06-05

**Authors:** Kaiqiong Zhao, Karim Oualkacha, Lajmi Lakhal‐Chaieb, Aurélie Labbe, Kathleen Klein, Antonio Ciampi, Marie Hudson, Inés Colmegna, Tomi Pastinen, Tieyuan Zhang, Denise Daley, Celia M.T. Greenwood

**Affiliations:** ^1^ Department of Epidemiology, Biostatistics and Occupational Health McGill University Montreal QC Canada; ^2^ Département de Mathématiques Université du Québec à Montrèal Montreal QC Canada; ^3^ Département de Mathématiques et de Statistique Université Laval Quebec City QC Canada; ^4^ Département des Sciences de la Décision HEC Montrèal Montreal QC Canada; ^5^ Lady Davis Institute for Medical Research Montreal QC Canada; ^6^ Department of Medicine McGill University Montreal QC Canada; ^7^ The Research Institute of the McGill University Health Centre Montreal QC Canada; ^8^ Center for Pediatric Genomic Medicine Children's Mercy Kansas City Kansas City MO USA; ^9^ Department of Psychiatry Douglas Mental Health University Institute McGill University Montreal QC Canada; ^10^ The Centre for Heart Lung Innovation, and Department of Medicine University of British Columbia Vancouver BC Canada; ^11^ Department of Human Genetics and Gerald Bronfman Department of Oncology McGill University Montreal QC Canada

**Keywords:** differentially methylated region, EM algorithm, generalized additive model, next‐generation sequencing, penalized regression splines

## Abstract

Identifying disease‐associated changes in DNA methylation can help us gain a better understanding of disease etiology. Bisulfite sequencing allows the generation of high‐throughput methylation profiles at single‐base resolution of DNA. However, optimally modeling and analyzing these sparse and discrete sequencing data is still very challenging due to variable read depth, missing data patterns, long‐range correlations, data errors, and confounding from cell type mixtures. We propose a regression‐based hierarchical model that allows covariate effects to vary smoothly along genomic positions and we have built a specialized EM algorithm, which explicitly allows for experimental errors and cell type mixtures, to make inference about smooth covariate effects in the model. Simulations show that the proposed method provides accurate estimates of covariate effects and captures the major underlying methylation patterns with excellent power. We also apply our method to analyze data from rheumatoid arthritis patients and controls. The method has been implemented in R package SOMNiBUS.

## 1 INTRODUCTION

Heritability is high for a wide range of human diseases (Maurano *et al*., 2012), but only a portion of it is attributable to additive genetic variation (Ober and Vercelli, 2011). Maher (2008) suggested that environmental exposures play an important role in explaining the “missing” heritability. Plausibly, such exposures, in interaction with genetic predisposition, may lead to epigenetic modification that alters gene regulation without changing genome sequence (Jaenisch and Bird, 2003). For example, differences in epigenetic profiles may explain how risk factors like age (Horvath, 2013) and smoking (Teschendorff *et al*., 2015) impact disease susceptibility. Consequently, examining how epigentic profiles contribute to disease development and are influenced by environmental factors can provide novel insights into disease etiology and possible therapies (Feinberg, 2007).

The most‐studied epigenetic mark is DNA methylation that primarily occurs at a cytosine‐guanine dinucleotide (ie, CpG site) (Lister *et al*., 2009). Localized differential methylation is a characteristic feature of many diseases, such as diabetes (Nilsson *et al*., 2014), Alzheimer's disease (De Jager *et al*., 2014) and autoimmune disorders (Liu *et al*., 2013).

Measuring large‐scale DNA methylation at single nucleotide resolution is now possible owing to the development of bisulfite sequencing protocols (Frommer *et al*., 1992), which can be implemented genome‐wide or in a set of targeted regions. Targeted Custom Capture Bisulfite Sequencing (TCCBS) platforms produce DNA methylation levels for comprehensive subsets of informative CpGs. Thus, epigenomic dysregulation can be captured at a much lower cost than whole‐genome bisulfite sequencing (WGBS). This approach's capacity to detect novel disease associations has been demonstrated (Allum *et al*., 2015; Li *et al*., 2015). In this work, we focus on analysis of predefined regions targeted by TCCBS, with the aim to identify differentially methylated regions (DMRs) that are associated with phenotypes or traits.

Methods for extracting interpretable results from the raw methylation data derived from either WGBS or TCCBS are greatly hindered by the variability in read depths, the many missing values, and the possibility of data errors. Specifically, due to the stochastic nature of sequencing and alignment, coverage—the total number of reads spanning a CpG site—varies substantially across sites and individual samples, which leads to wide‐ranging precision for methylation proportions, and to many missing values. In fact, estimates of DNA methylation are correlated with read depths (Stephens *et al*., 2016). Furthermore, the observed counts of methylated and unmethylated reads could be contaminated by errors arising from excessive or insufficient bisulfite treatment, and from misalignment of reads or other aspects of the sequencing processes. Studies show that ignoring these errors could bias inference about the associations of interest (Cheng and Zhu, 2013; Lakhal‐Chaieb *et al*., 2017).

Additionally, due to cell type specific differences in methylation levels, variability in cell type mixture proportions has a strong effect on observed levels of methylation from mixed tissue samples. This mixture, as well as factors known to alter methylation levels, such as age (Horvath, 2013), can confound associations of interest. Hence, it is essential to develop methods to adjust methylation signals for multiple covariates.

Moving in this direction, approaches have been proposed for identifying DMRs from bisulfite sequencing data; see overviews in Shafi *et al*. (2017) and Yu and Sun (2016a). Typically, to account for spatial correlations of methylation between neighboring CpG sites, strategies include Hidden Markov models (HMM) (Sun and Yu, 2016; Yu and Sun, 2016b; Shokoohi *et al*., 2018), hierarchical models with autoregressive or random walk correlation structures (Rackham *et al*., 2017; Korthauer *et al*., 2019), and kernel‐based smoothing methods (Hansen *et al*., 2012; Hebestreit *et al*., 2013; Lakhal‐Chaieb *et al*., 2017). However, none of these methods meet all the desirable objectives *simultaneously*: regional testing, estimation of multiple covariate effects, adjustment for read depth variability, and experimental errors. For example, several of the current HMM‐based (Sun and Yu, 2016; Yu and Sun, 2016b) and hierarchical methods (Rackham *et al*., 2017) only test for differential methylation between two independent groups of samples and do not allow for the adjustment of multiple covariates. Approaches using a binomial mixed model for DNA methylation analysis (Lea *et al*., 2015; Weissbrod *et al*., 2017) allow for multiple covariates and can capture sample correlations, but were only designed for single site analysis. BSmooth (Hansen *et al*., 2012), a kernel‐based method, detects differential methylation after converting the methylated and total counts to proportions. However, this conversion could lead to reduced power since it disregards read depth variability and fails to distinguish between noisy and accurate measurements (Rackham *et al*., 2017). Moreover, most of the existing methods ignore experimental errors. On the other hand, the only approach accounting for data errors, the Smooth Methylation Status Call (SMSC) (Lakhal‐Chaieb *et al*., 2017), is only developed for data from a single cell type.

More importantly, most of the existing methods are of a two‐stage nature (Hansen *et al*., 2012; Hebestreit *et al*., 2013; Lakhal‐Chaieb *et al*., 2017). Typically, they first smooth the raw methylation data for each sample separately, and then, in the second stage, they estimate covariate effects by modeling the smoothed methylation data. These per‐sample smoothing strategies do not take advantage of information contained across samples and fail to fully exploit the fact that samples with similar covariate profiles (eg, disease status, cell type composition, or other phenotypes of interest) can be expected to share similar methylation patterns. In addition, separating smoothing and inference steps results in biased uncertainty estimates. In summary, it would be highly desirable to develop a general framework of analysis, which collapses smoothing and testing steps into a single step, and simultaneously addresses regional testing, estimation of multiple covariate effects, adjustment for read depth variability, and experimental errors.

In this paper, we propose such a general framework. Our strategy allows information to be shared not only between nearby CpGs, but also across samples, thus providing greater sensitivity for capturing patterns common to several samples of similar characteristics (rather than one sample).

Specifically, our approach is built on a hierarchical regression model that describes bisulfite sequencing data. We assume, as in Lakhal‐Chaieb *et al*. (2017) and Cheng and Zhu (2013), that the observed read counts arise from an unobserved latent true methylation state compounded by errors. These true methylation counts are then modeled by a binomial distribution, dependent on read depth. Note that the probability parameter of this binomial distribution depends on the sample‐level covariates of interest, such as cell‐type mixture proportions and the trait of interest, but also nearby methylation information. To capture realistic methylation patterns across regions, we additionally allow baseline methylation levels, covariate effects, and adjustment effects to vary smoothly along genomic positions: this is done by using spines. This amounts to borrowing information from the local correlation structures between methylation levels, and allows us to remedy local information gaps due to missingness. This formulation naturally allows for any number of covariates in the model.

This article is organized as follows. Section 2 describes the proposed model along with its estimation and inference procedures. A motivating data example from a study of cases with rheumatoid arthritis (RA) and controls is described in Section 3. Simulation studies evaluating the performance of our proposed method and comparing our type I errors and power to existing methods are summarized in Section 4. The paper concludes with a discussion in Section 5.

## 2 METHOD

### 2.1 Notation and data

We consider DNA methylation measures over a targeted genomic region from *N* independent samples. Let $m_{i}$ be the number of CpG sites for the ith sample, i=1,2,…N. We write $t_{ij}$ for the genomic position (in base pairs) for the ith sample at the jth CpG site, $j=1,2,\ldots ,m_{i}$. The set of genomic positions captured in different samples do not have to be identical because each sample has an individual profile of covered CpG sites, due to read depth variability. Methylation levels at a site are quantified by the number of methylated reads and the total number of reads. We define $X_{ij}$ as the total number of reads aligned to CpG *j* from sample *i*. The tissue samples sent for bisulfite sequencing experiments from most studies will normally be composed of a mixture of cell types. For example, common cell types are in blood: granulocytes, T cells, B cells, monocytes, neutrophils, and eosinophils; in adipose tissues: adipocyte, preadipocyte, endothelial and mural cells. Thus, the reads obtained at a CpG site are likely to capture contributions from different cell types; the true underlying methylation statuses are probably different across these $X_{ij}$ reads. We denote the *true* methylation status for the kth read obtained at CpG *j* of sample *i* as $S_{ijk}$, where $k=1,2,\ldots X_{ij}$. $S_{ijk}$ is binary and we define $S_{ijk}=1$ if the corresponding read is methylated and $S_{ijk}=0$ otherwise. In the presence of experimental errors in sequencing or preprocessing, the *observed* methylation status, written as $Y_{ijk}$, can be distinct from the true underlying information $S_{ijk}$. We denote $Y_{ijk}=1$ if the corresponding read is observed as methylated and $Y_{ijk}=0$ otherwise. We additionally denote the *true* and *observed methylated counts* at CpG *j* for sample *i* with $S_{ij}=\sum_{k=1}^{X_{ij}}S_{ijk}$ and $Y_{ij}=\sum_{k=1}^{X_{ij}}Y_{ijk}$, respectively. Furthermore, we assume that we have the information on *P* covariates for the *N* samples, denoted as $Z_{i}=\left(Z_{1i},Z_{2i},\ldots Z_{Pi}\right)$, for i=1,2,…N.

### 2.2 Model

We built here on concepts introduced in Cheng and Zhu (2013) and Lakhal‐Chaieb *et al*. (2017) to account for experimental errors. We assume that, depending on the true underlying methylation status $S_{ijk}$, the observed status $Y_{ijk}$ is a Bernoulli variable with parameters *p*_0_ or *p*_1_, that is,

(1) $$\begin{array}{ccc} p_{0} & = & P(Y_{ijk}=1|S_{ijk}=0),\\ p_{1} & = & P(Y_{ijk}=1|S_{ijk}=1). \end{array}$$

Here, these two parameters capture errors; *p*_0_ is the rate of false methylation calls, and $1-p_{1}$ is the rate of false nonmethylation calls. These rates are assumed to be constant across all reads and positions. The error parameters *p*_0_ and *p*_1_ can be estimated by looking at raw sequencing data at CpG sites known in advance to be methylated or unmethylated (Wreczycka *et al*., 2017). We assume hereafter that *p*_0_ and *p*_1_ are known. Implications of such an assumption is discussed later in the Supporting Information Section 2.2.

We then assume the true methylated counts $S_{ij}$ follows a binomial distribution with a methylation proportion parameter $\pi_{ij}$ that depends on the sample‐level covariates ***Z_i_***, and on nearby methylation patterns. Specifically,

(2) $$\begin{array}{ccc} S_{ij}|Z_{i},X_{ij} & ∼ & \text{Binomial}(X_{ij},\pi_{ij}),\\ g(\pi_{ij}) & = & \beta_{0}\left(t_{ij}\right)+\sum_{p=1}^{P}\beta_{p}\left(t_{ij}\right)Z_{pi}, \end{array}$$

where g(·) is a logit link function and $\beta_{0}\left(t_{ij}\right)$ and ${\left\{\beta_{p}\left(t_{ij}\right)\right\}}_{p=1}^{P}$ are functional parameters for the intercept and covariate effects. This amounts to assuming smoothly varying methylation levels and covariate effects on methylation levels across our targeted small genomic regions. In practice, to estimate Model (2), the functions $\beta_{p}\left(t_{ij}\right)$ can be represented by the coefficients of a chosen spline bases of rank $L_{p}$,

$$\beta_{p}\left(t_{ij}\right)=\sum_{l=1}^{L_{p}}\alpha_{pl}B_{l}^{\left(p\right)}\left(t_{ij}\right),\;\text{for}\;p=0,1,\ldots P,$$

where ${\left\{B_{l}^{\left(p\right)}\left(\cdot \right)\right\}}_{l=1}^{L_{p}}$ denotes the spline basis, and $\alpha_{p}={\left(\alpha_{p1},\ldots \alpha_{pL_{p}}\right)}^{T}\in R^{L_{p}}$ are the coefficients to be estimated. In this way, model (2) becomes a generalized linear model (GLM), g(π)=Xα, where $\pi ={\left(\pi_{11},\ldots \pi_{1m_{1}},\pi_{21},\ldots \pi_{2m_{2}},\ldots \pi_{Nm_{N}}\right)}^{T}\in {\left[0,1\right]}^{M}$ with $M=\sum_{i=1}^{N}m_{i}$, $\alpha \in R^{K}$ with $K=\sum_{p=0}^{P}L_{p}$, and X is the spanned design matrix of dimension M×K, stacked with elements $B_{l}^{\left(p\right)}\left(t_{ij}\right)\times Z_{pi}$; for detailed forms, see Supporting Information Appendix A.

To avoid over‐fitting, we penalize departure from smoothness, using penalized regression splines (Wahba, 1980; Parker and Rice, 1985). Specifically, we use a comparatively large number of knots (equivalent to large $L_{p}$) and a penalization, quantified by the integrated squared curvature of the splines, is added as an extra term in the log‐likelihood function (loss function),

(3) $$L^{\mathrm{Penalization}}=\sum_{p=0}^{P}\lambda_{p}\int {\left(\beta_{p}^{\prime \prime }\left(t\right)\right)}^{2}dt=\sum_{p=0}^{P}\lambda_{p}{\alpha_{p}}^{T}A_{p}\alpha_{p}.$$

In Equation (3), ${A_{p}}^{\prime }s$ are $L_{p}\times L_{p}$ positive semidefinite matrices with the $\left(l,l^{\prime }\right)$ element $A_{p}\left(l,l^{\prime }\right)=\int {B^{\left(p\right)}}_{l}^{\prime \prime }\left(t\right){B^{\left(p\right)}}_{l^{\prime }}^{\prime \prime }\left(t\right)dt;$ these are fixed quantities given the specified set of basis functions. The weights $\lambda_{p}$, that is, the smoothing parameters, are positive parameters that establish a trade‐off between the closeness of the curve to the data and the smoothness of the fitted curves. Note that there is one smoothing parameter per covariate in our model. The smoothing process across targeted regions is accomplished by adding the penalization terms in Equation (3) to the model in Equation (2).

### 2.3 Estimation

#### 2.3.1 Penalized complete likelihood

If the true methylated counts $S_{ij}$ were available, model (2) with penalization (3) would be estimated by maximizing the penalized log‐likelihood,

$$\begin{array}{ccc} l^{\mathrm{complete}}\left(S;\alpha ,\lambda \right) & = & l\left(S;\alpha \right)-\frac{1}{2}\sum_{p=0}^{P}\lambda_{p}\alpha_{p}^{T}A_{p}\alpha_{p}\\ & = & l\left(S;\alpha \right)-\frac{1}{2}\alpha^{T}A_{\lambda }\alpha , \end{array}$$

where $l\left(S;\alpha \right)\;=\;\sum_{i=1}^{N}\sum_{j=1}^{m_{i}}\left\{S_{ij}\log\left(\pi_{ij}\right)\;+\;\left(X_{ij}\;-\;S_{ij}\right)\log\left(1-\pi_{ij}\right)\right\}$, and ***A_λ_*** is a K×K positive semidefinite block diagonal matrix of the form $A_{\lambda }=\text{Diag}\left\{\lambda_{0}A_{0},\lambda_{1}A_{1},\ldots ,\lambda_{P}A_{P}\right\}$. This is also the complete‐data log‐likelihood of the joint distribution of ***Y*** and ***S***, that is, log(f(S))+log(f(Y∣S)); indeed, f(Y∣S) only depends on the known error rates *p*_0_ and *p*_1_, and bears no information on the parameters of interest.

#### 2.3.2 Smoothed E‐M algorithm

In practice, the true methylation data, $S_{ij}$, are unknown and one only observes $Y_{ij}$, which is a mixture of binomial counts arising from both the truly methylated and truly unmethylated reads. The EM algorithm (Dempster *et al*., 1977) allows us to estimate model (2) based on the observed data $Y_{ij}$, by repeatedly replacing a trial estimate $\left(\alpha^{★},\lambda^{★}\right)$ by a new (α,λ), which is a maximum of the function

(4) $$\begin{array}{ccc} Q(\alpha |\alpha^{★}) & = & E\left\{l^{\mathrm{complete}}\left(S;\alpha ,\lambda \right)|Y,\alpha^{★}\right\}\\ & = & l\left(\eta^{★};\alpha \right)-\frac{1}{2}\alpha^{T}A_{\lambda }\alpha . \end{array}$$

*E step* In Equation (4) $\eta^{★}={\left(\eta_{11}^{★},\ldots ,\eta_{1m_{1}}^{★},\eta_{21}^{★},\ldots ,\eta_{2m_{2}^{★}},\ldots ,\eta_{Nm_{N}}^{★}\right)}^{T}\in R^{M}$ are conditional expectations of $S_{ij}$ given $Y_{ij}$ evaluated at the trial estimates $\left(\alpha^{★},\lambda^{★}\right)$; in our case, these take the form

(5) $$\begin{array}{ccc} \eta_{ij}^{★} & = & E\left(S_{ij}|Y_{ij};\alpha^{★},\lambda^{★}\right)=\frac{Y_{ij}p_{1}\pi_{ij}^{★}}{p_{1}\pi_{ij}^{★}+p_{0}\left(1-\pi_{ij}^{★}\right)}\\ & & +\;\frac{\left(X_{ij}-Y_{ij}\right)\left(1-p_{1}\right)\pi_{ij}^{★}}{\left(1-p_{1}\right)\pi_{ij}^{★}+\left(1-p_{0}\right)\left(1-\pi_{ij}^{★}\right)}, \end{array}$$

with $\pi_{ij}^{★}=g^{-1}\left(X\alpha^{★}\right)$, which depends on $\lambda^{★}$ via the dependence of $\alpha^{★}$ on $\lambda^{★}$. Calculating these conditional expectations $\eta_{ij}^{★}$ from (5) constitutes the E step of our algorithm.

*M step* Each M step involves maximizing the Q function in (4) to update ***α*** and ***λ***. This is a penalized (GLM) likelihood maximization problem with multiple quadratic penalties, previously studied in Wood (2011), Wood *et al*. (2016), and Wood and Fasiolo (2017). Our computational strategy for estimating smoothing parameters ***λ*** is a nested optimization procedure (Wood, 2011), with an outer iteration for optimizing ***λ*** and an inner penalized iteratively reweighted least squares (P‐IRLS) iteration to estimate ***α*** given the trial value of ***λ*** from the outer iteration.

For given values of smoothing parameters $\lambda =(\lambda_{0},\lambda_{1},\ldots \lambda_{P})$, a unique maximizer of expression (4) is readily computed by P‐IRLS; see more details in the Supporting Information Appendix B. Specifically, the outer iteration involves maximizing a restricted likelihood for smoothing parameters ***λ***, which is obtained by integrating ***α*** out of the joint likelihood for ***λ*** and ***α***. We rely on the work done by Wood (2011) and use a Laplace approximated restricted likelihood; see more details in the Supporting Information Appendix C. As the analytical forms for derivatives and Hessians of this restricted likelihood are also available, the optimization for ***λ*** in the outer iteration can be readily achieved via Newton's method.

Although the combination is undoubtedly computationally complex, the nested iterations will guarantee convergence for models with properly defined likelihoods (Wood, 2011; Wood *et al*., 2016).

*E‐M iteration* We iterate between the E and M steps until convergence to obtain $\hat{\alpha }$ and $\hat{\lambda }$. Given the estimates of basis coefficients $\alpha_{p}$, for p=0,1,…P, the functional parameters $\beta_{p}\left(t\right)$ can be thus estimated by $\hat{\beta_{p}\left(t\right)}={\left\{B^{\left(p\right)}\left(t\right)\right\}}^{T}\left\{\hat{\alpha_{p}}\right\}$, where *t* is a genomic position lying within the range of the input positions $\left\{t_{ij}\right\}$, and $B^{\left(p\right)}\left(t\right)={\left(B_{1}^{\left(p\right)}\left(t\right),B_{2}^{\left(p\right)}\left(t\right),\ldots B_{L_{p}}^{\left(p\right)}\left(t\right)\right)}^{T}\in R^{L_{p}}$ is a column vector with nonrandom quantities obtained from evaluating the set of basis functions ${\left\{B_{l}^{\left(p\right)}\left(\cdot \right)\right\}}_{l}$ at position *t*.

### 2.4 Inference for smooth covariate effects

To obtain a quantification of the uncertainty accompanying the smoothed EM estimates for the covariate effects $\left\{\beta_{1}\left(t\right),\beta_{2}\left(t\right),\ldots \beta_{P}\left(t\right)\right\}$, we additionally estimate their pointwise confidence intervals (CI) in Section 2.4.1, and obtain tests of hypotheses for these effects in Section 2.4.2. This inference is carried out conditional on the values of smoothing parameter ***λ***; that is, the uncertainty in estimating λ is not accounted for. The potential bias associated with this assumption is shown to be small; see the pointwise confidence interval coverage in Figure 4 and the distribution of region‐based *P*‐values under the null in Figure 5.

#### 2.4.1 Confidence interval estimation

Analytical derivation for standard errors usually involves calculating the observed Fisher information for parameters ***α*** from the marginal log‐likelihood for ***Y***. However, in this case, a direct calculation of the observed Fisher information is analytically intractable because the observed ***Y*** follows a mixture of two binomial distributions. To circumvent this problem, we rely on the work of Louis (1982) and Oakes (1999), which showed that this Fisher information can be calculated solely from the *Q* function (4), without referring to the marginal distribution of ***Y***.

Theorem 1 Under the usual regularity conditions for maximum likelihood, we have the following asymptotic results for the estimators $\hat{\alpha }$ obtained from the smoothed‐EM algorithm,

$$\sqrt{M}\left(\hat{\alpha }-\alpha \right)\overset{L}{⟶}{\mathrm{MVN}}_{K}\left(0,I^{-1}\right),\;\;\text{as}\;M\to \infty .$$

Here, *K* is the dimension of the spline coefficients ***α***, and $I=E[-H_{ij}\left(\alpha \right)]$. Specifically $H_{ij}\left(\alpha \right)$ has the form

$$H_{ij}\left(\alpha \right)=X_{\left(l,\right)}^{T}\left(-X_{ij}w_{ij}+\delta_{ij}w_{ij}\right)X_{\left(l,\right)}-A_{\lambda },$$

where $X_{\left(l,\right)}$ is the lth row of the design matrix X, which corresponds to the CpG *j* of sample *i*, $w_{ij}=\pi_{ij}\left(1-\pi_{ij}\right)$ is the element of the weight matrix, and

$$\begin{array}{ccc} \delta_{ij} & = & \frac{Y_{ij}p_{1}p_{0}}{{\left[p_{1}\pi_{ij}+p_{0}\left(1-\pi_{ij}\right)\right]}^{2}}\\ & & +\;\frac{\left(X_{ij}-Y_{ij}\right)\left(1-p_{1}\right)\left(1-p_{0}\right)}{{\left[\left(1-p_{1}\right)\pi_{ij}+\left(1-p_{0}\right)\left(1-\pi_{ij}\right)\right]}^{2}}. \end{array}$$

The proof of Theorem 1 is given in the Supporting Information Appendix D. Theorem 1 provides the desired variance‐covariance matrix of the EM estimators $\hat{\alpha }$, which can be estimated using the observed Fisher information

$$\hat{V\text{ar}}\left(\hat{\alpha }\right)={\left\{-H(\hat{\alpha })\right\}}^{-1},$$

where $H\left(\hat{\alpha }\right)=\sum_{i,j}H_{ij}\left(\hat{\alpha }\right)$. Let ***V*** denote this variance estimator and ***V_p_*** be the diagonal blocks of ***V*** corresponding to $\alpha_{p}$, with dimensions $L_{p}\times L_{p}$. As β(t) is a linear combination of coefficients ***α_p_***, the estimated variance of $\hat{\beta_{p}\left(t\right)}$ takes the form $\hat{V\text{ar}}\left(\hat{\beta_{p}\left(t\right)}\right)={\left\{B^{\left(p\right)}\left(t\right)\right\}}^{T}V_{p}\left\{B^{\left(p\right)}\left(t\right)\right\}$. Therefore, the confidence interval for $\beta_{p}\left(t\right)$ at significance level ν can be estimated by $\hat{\beta_{p}\left(t\right)}\pm Z_{\nu /2}\sqrt{\hat{V\text{ar}}\left(\hat{\beta_{p}\left(t\right)}\right)}$, for any *t* in the range of interest, where $Z_{\nu /2}$ is ν/2 (upper‐tail) quantile of a standard normal distribution.

#### 2.4.2 Hypothesis testing for a regional zero effect

We can also construct a region‐wide test of the null hypothesis

$$H_{0}:\beta_{p}\left(t\right)=0,\text{for}\;\text{any}\;t\;\text{in}\;\text{the}\;\text{genomic}\;\text{interval}.$$

This test depends on the association between covariate $Z_{p}$ and methylation levels across the region, after adjustment for all the other covariates, and the null hypothesis is equivalent to $H_{0}:\alpha_{p}=0$. A Wald‐type statistic can be naturally proposed as

$$T_{p}={\hat{\alpha_{p}}}^{T}{\left\{V_{p}\right\}}^{-1}\hat{\alpha_{p}},$$

where ${\left\{V_{p}\right\}}^{-1}$ denotes inverse if ***V_p_*** is nonsigular; for singular ***V***_*p*_, the inverse is replaced by the Moore‐Penrose inverse ${\left\{V_{p}\right\}}^{-}$. If ***α_p_*** is a vector of unpenalized coefficients, under the null hypothesis, $T_{p}$ asymptotically follows a Chi‐square distribution with degrees of freedom $L_{p}$. In the presence of smoothness penalization, $L_{p}$ should be replaced by the effective degrees of freedom (EDF), $\tau_{p}$, which depends on the magnitude of smoothing parameter $\lambda_{p}$ and is smaller than $L_{p}$. Motivated by the work of Wood (2013), we define the EDF $\tau_{p}$ as

(6) $$\tau_{p}=\sum_{l=a_{p}}^{b_{p}}{\left(2F-FF^{T}\right)}_{\left(l,l\right)},\;\text{for}\;p=0,1,\ldots P,$$

where $a_{p}=\sum_{m=0}^{p-1}L_{m}+1$ if p>0 and $a_{p}=1$ if p=0, $b_{p}=\sum_{m=0}^{p}L_{m}$ for any *p*, and ${\left(•\right)}_{\left(l,l\right)}$ stands for the lth leading diagonal element of a matrix. In (6), ***F*** is the smoothing matrix of our model, which has the form $F={\left(X^{T}\hat{W}X+A_{\hat{\lambda }}\right)}^{-1}X^{T}\hat{W}X$, where $\hat{W}$ is the weight matrix whose diagonal is $X_{ij}\;\hat{\pi_{ij}}\left(1-\hat{\pi_{ij}}\right)$. A joint null hypothesis that evaluates the effects of multiple covariates can be defined in a similar way.

Hereafter we refer the proposed novel method including the region‐wide test and the smooth covariate estimation as SOMNiBUS (SmOoth ModeliNg of BisUlfite Sequencing).

## 3 METHYLATION DATA FROM AN RA STUDY

To illustrate our method, we report our analysis on data from an RH study (Hudson *et al*., 2017). Methylation profiles of cell‐separated blood samples of 22 rheumatoid arthritis (RA) patients and 21 healthy individuals were measured with custom captured targeted bisulfite sequencing. We focus on one targeted region on chromosome 4 near gene *BANK1*, which is known to show cell‐type‐specific methylation levels (Hillier *et al*., 2005). Three additional targeted regions from the same data set are also analyzed in the Supporting Information Section 3. In this *BANK1* region, methylation levels are available at 123 CpG sites. There are 25 samples from circulating T cells and 18 samples from monocytes. We consider two binary covariates—RA status and cell type—and study their impact on methylation pattern in this region.

**FIGURE 1:**
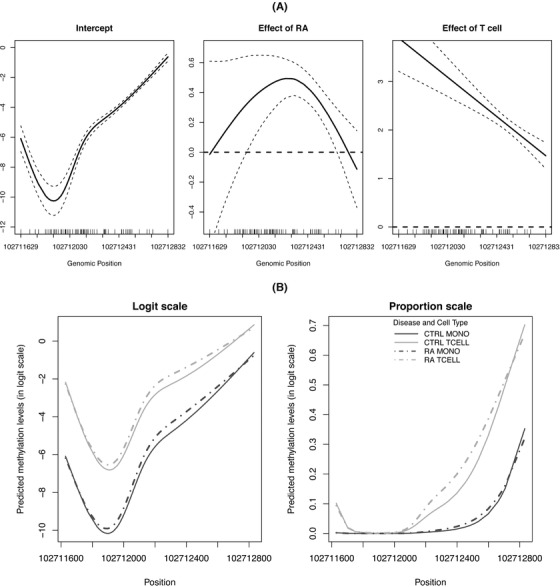
(A), The estimates (solid lines) and 95% pointwise confidence intervals (dashed lines) of the intercept, the smooth effect of RA and cell type (T cells versus monocytes) on methylation levels. (B), The predicted methylation levels in the logit scale (left) and proportion scale (right) for the four groups of samples with different disease and cell type status. The region‐based *P*‐values for the effect of RA status and T cell type are calculated as 1.11E−16 and 6.37E−218, respectively

To fit SOMNiBUS, we specified error parameters $p_{0}=0.003$ and $1-p_{1}=0.1$; the value 0.003 was reported by Prochenka *et al*. (2015) as insufficient conversion rate and 0.1 was estimated as the average excessive conversion rate in our data using the method proposed by Lakhal‐Chaieb *et al*. (2017). We used cubic splines of rank $L_{p}=5$ to expand the smooth terms in the model. Figure 1A shows the estimated smooth covariate effects on methylation levels in the targeted *BANK1* region. The panel “Intercept” displays the methylation pattern (on the logit scale) for control samples with the monocyte cell type. The panel “Effect of RA” displays the pattern of methylation difference (on the logit scale) between RA samples and control samples with the same cell type. This figure suggests that RA patients show slightly higher methylation levels in the middle part of the region, compared to controls. The panel “Effect of Tcell” represents the difference of methylation levels (on the logit scale) between T cell samples and monocyte samples with the same disease status. This effect curve, along with the confidence interval bands, clearly shows a highly significant increase of methylation in T cells relative to monocytes. Figure 1B displays the predicted methylation proportions in the four groups of samples, defined by cell type and RA status. Overall, Figure 1 demonstrates the smoothness of the fits, the ability to use multiple covariates simultaneously, and the ease of interpretation of results across the region. Region‐wide tests of significance for the two covariates are highly significant (Figure 1). We also applied five alternative methods, described in Section 4; see Table S3 in the Supporting Information.

## 4 SIMULATION STUDY

We conducted simulation to (a) demonstrate that the proposed inference of smooth covariate effects is valid, and to (b) compare the performance of our method with five existing methods: BiSeq (Hebestreit *et al*., 2013), BSmooth (Hansen *et al*., 2012), SMSC (Lakhal‐Chaieb *et al*., 2017), dmrseq (Korthauer *et al*., 2019) and GlobalTest (Goeman *et al*., 2006), in terms of type I error and power. The first three methods are typical examples of two‐stage analytic approaches. In the first stage, kernel smoothing (local likelihood estimation) is applied to the methylation data of each sample separately. In the second stage, the smoothed methylation data are further analyzed. Specifically, BiSeq calculates the average of Wald statistics from single‐site beta regression models, while BSmooth and SMSC calculate the sum of *t*‐statistics across loci; these statistics are used to test for differential methylation of a region. In contrast, dmrseq and GlobalTest are one‐stage approaches that fit their models directly to the raw methylation proportions in a region. Specifically, dmrseq assesses the strength of the covariate effect using a Wald test statistic within a generalized least square regression model, whereas GlobalTest uses an improved score test in a linear regression model.

Notably, like SOMNiBUS, both GlobalTest and BiSeq are primarily tailored to targeted bisulfite sequencing data with previously identified regions, whereas BSmooth, SMSC and dmrseq are designed for WGBS data. Specifically, BSmooth and SMSC define DMRs at adjacent CpG sites with absolute t‐statistics above a defined threshold. The final product from the original software of BSmooth is a list of DMRs that are ranked by the sum of t‐statistics; however, BSmooth does not provide region‐based *P*‐values. To allow comparisons with SOMNiBUS, we estimated the empirical regional *P*‐values for BSmooth by permuting the values of the covariate of interest 1000 times. When analyzing WGBS data, dmrseq first constructs candidate regions based on a user‐defined cutoff of the smoothed methylation proportion differences, and then fits a generalized least squares regression model with autoregressive error structure to the transformed methylation proportions. Furthermore, the inference inside dmrseq is drawn from permutations—its approximate null distribution is generated by pooling a set of region‐level statistics of many candidate regions from all permutations. To better adapt dmrseq to a single targeted region: (i) we used a small cutoff of methylation differences (1E−5) for detecting candidate (sub)regions, which ensures fewer CpG sites to be filtered out; (ii) we applied a relatively large number of permutations (B=500) to generate a null distribution of test statistics; (iii) we reported the raw *P*‐values without the multiplicity corrections. Note that in some simulations, dmrseq reported more than one DMR in the region. Therefore, for a fairer comparison, we calculated the dmrseq's *P*‐value as the minimum over the reported chunks' *P*‐values.

Among the five competitive methods, dmrseq, GlobalTest, and BiSeq allow adjustment for multiple covariates. SMSC is the only approach accounting for experimental errors; however, it is conceptually restricted to data from a single cell type.

### 4.1 Simulation design

Our simulation design is inspired by the data example described in Section 3. Methylation regions of the same size and with the same CpG distribution as the *BANK1* region were simulated under various settings. We first generated the read depth $X_{ij}$ by resampling with replacement the read depth values from the real data. To specify covariates $Z_{p}$ and their effect curves $\beta_{p}\left(t\right)$, we then considered the following two scenarios.

#### 4.1.1 Scenario 1 – Multiple covariates

In this case, P=3 binary covariates $Z_{1},Z_{2}$, and *Z*_3_ were generated independently for each sample. *Z*_1_ and *Z*_2_ were simulated from Bernoulli distributions with proportions 0.51 and 0.58, which were the proportions of RA and T cell samples in the RA data set. The functional parameters for intercept and covariate effects, $\beta_{0}\left(t\right)$, $\beta_{1}\left(t\right)$, and $\beta_{2}\left(t\right)$, were specified to have the same shapes as seen in the *BANK1* region (Figure 1A). Covariate *Z*_3_ was generated from a Bernoulli distribution with proportion parameter 0.5 and had zero effect on methylation, that is, $\beta_{3}\left(t\right)=0$, for all *t* in the region. The inference results for the effect of the null covariate, *Z*_3_, provide information on type I error.

#### 4.1.2 Scenario 2 – Single covariate

**FIGURE 2:**
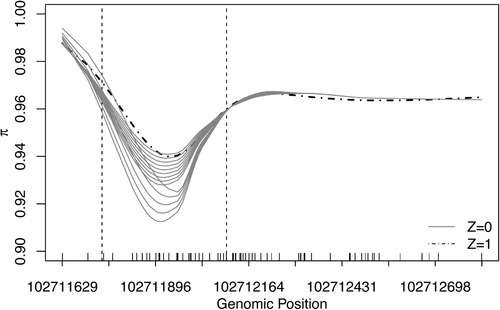
The 14 simulation settings of methylation parameters π(t) in Scenario 2. Methylation parameters for samples with Z=1 (dotted‐dashed black curve) are fixed across settings, whereas the methylation parameters for samples from group Z=0 (solid gray lines) vary across simulations corresponding to different degrees of closeness between methylation patterns in the two groups

We also considered the case of a single binary covariate (P=1), generated from Bernoulli (0.5), with a variety of regional effect curves. The forms of the functional parameters $\beta_{0}\left(t\right)$ and $\beta_{1}\left(t\right)$ were specified to yield methylation proportion parameters $\pi_{0}\left(t\right)$ and $\pi_{1}\left(t\right)$ as depicted in Figure 2, where $\pi_{0}\left(t\right)$ and $\pi_{1}\left(t\right)$ denote the methylation parameters for samples with Z=0 and Z=1 at position *t*. As shown in Figure 2, these 14 settings of $\pi_{0}\left(t\right)$ correspond to varying levels of closeness between methylation patterns from the two groups.The corresponding values of $\beta_{0}\left(t\right)$ and $\beta_{1}\left(t\right)$ under these 14 settings are shown in the Supporting Information Figure S1. We defined the maximum deviation as the maximum difference between $\pi_{1}\left(t\right)$ and $\pi_{0}\left(t\right)$, for *t* in the section indicated by the dashed lines in Figure 2, where the curves of π_1_ and π_0_ mainly differ. Simulation scenario 2 is aimed at investigating the power for detecting DMRs at varying levels of maximum derivations.

Given the values of $\left\{Z_{1},\ldots Z_{P}\right\}$ and $\left\{\beta_{p}\left(t\right),p=0,1,\ldots P\right\}$ under each setting, the true methylation counts $S_{ij}$ were simulated from the model specified in (2). We then generated the observed methylated counts $Y_{ij}$ according to Equation (1), which implies

$$Y_{ij}|S_{ij}∼\text{Binomial}\left(S_{ij},p_{1}\right)+\text{Binomial}\left(X_{ij}-S_{ij},p_{0}\right).$$

We considered two settings for error parameters *p*_0_ and *p*_1_: (1) $p_{0}=0.003$ and $1-p_{1}=0.1$, and (2) $p_{0}=1-p_{1}=0$.

Under each scenario and setting, we generated data sets with sample sizes N=40,100,150 and 400, each 1000 times. We then applied SOMNiBUS along with methods BiSeq, dmrseq, BSmooth, SMSC, and GlobalTest to the simulated data sets. Unless otherwise stated, default settings were used for the five alternative methods. For our approach SOMNiBUS, we used cubic splines with dimension $L_{p}=5$ to parameterize the smooth terms of interest. We also assumed that the correct values of error parameters *p*_0_ and *p*_1_ were known, although we conducted sensitivity analyses to this assumption (see Discussion and Supporting Information Section 2.2 ). All simulation parameters are summarized in the Supporting Information Table S1.

### 4.2 Simulation results

**FIGURE 3:**
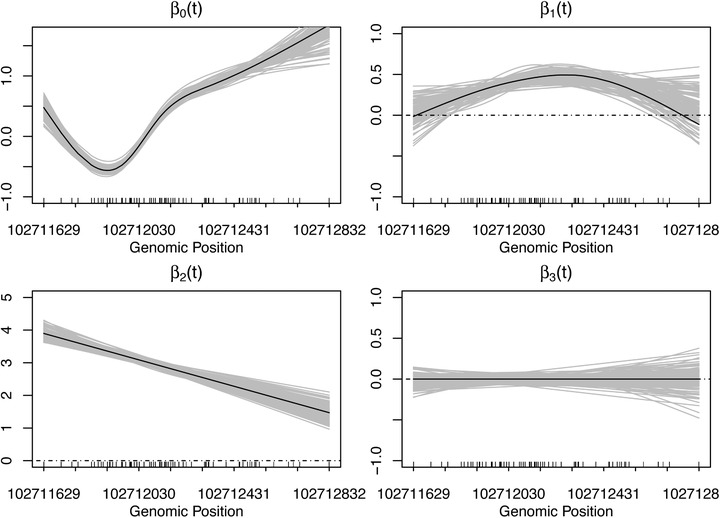
Estimates of smooth covariate effects (gray) over the 100 simulations in Scenario 1, using SOMNiBUS. The black curves are the true functional parameters used to generate the data. Data with sample size N=40 were generated with error

Figure 3 presents the estimates of the functional parameters $\beta_{0}\left(t\right),\beta_{1}\left(t\right),\beta_{2}\left(t\right)$ and $\beta_{3}\left(t\right)$ over 100 simulations, obtained from SOMNiBUS; here, data were generated under Scenario 1, with sample size N=40 and error parameters $p_{0}=0.003$ and $1-p_{1}=0.1$. It demonstrates that the proposed method provides unbiased curve estimates for all the four functional parameters in the model, and it can correctly capture both linear and nonlinear smooth covariate effects.

**FIGURE 4:**
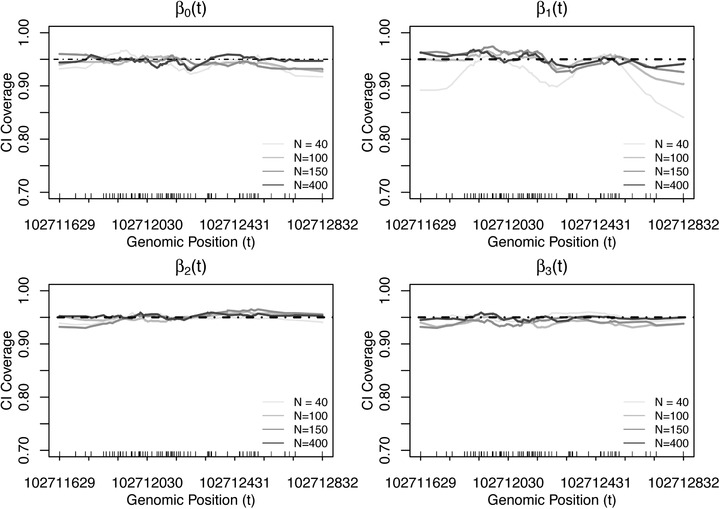
Coverage probability of confidence intervals over 1000 simulations under different sample sizes (N=40,100,150,400). Data were generated with error, under simulation Scenario 1

Figure 4 displays the empirical coverage probabilities of CIs over 1000 simulations of Scenario 1. The empirical coverage probabilities are defined as the percentage of simulations where the analytical 95% confidence interval (proposed in Section 2.4.1) covers the true value of the parameter. Overall, the coverage probabilities for $\beta_{2}\left(t\right)$ and $\beta_{3}\left(t\right)$ with linear shapes are closer to the nominal level 95% than the two nonlinear shapes for $\beta_{0}\left(t\right)$ and $\beta_{1}\left(t\right)$. This result can be expected, because nonlinear patterns require more parameters, which leads to less accurate inference results than linear patterns, given the same amount of information. When sample size is 40, the coverages for $\beta_{1}\left(t\right)$ tend to be less than 95%, especially at the boundaries. This may be because $\beta_{1}\left(t\right)$ has a nonlinear shape with relatively small effect sizes across the region, which poses extra difficulties in estimation compared to the shapes that are away from the null, such as $\beta_{0}\left(t\right)$. In summary, Figure 4 shows that the coverages of our 95% confidence intervals attain their nominal values in most of the simulation settings. This suggests that the proposed CI estimation approach quantifies the underlying uncertainty in the smoothed‐EM estimates with reasonable accuracy, although it ignores the uncertainty from estimating the smoothing parameters.

**FIGURE 5:**
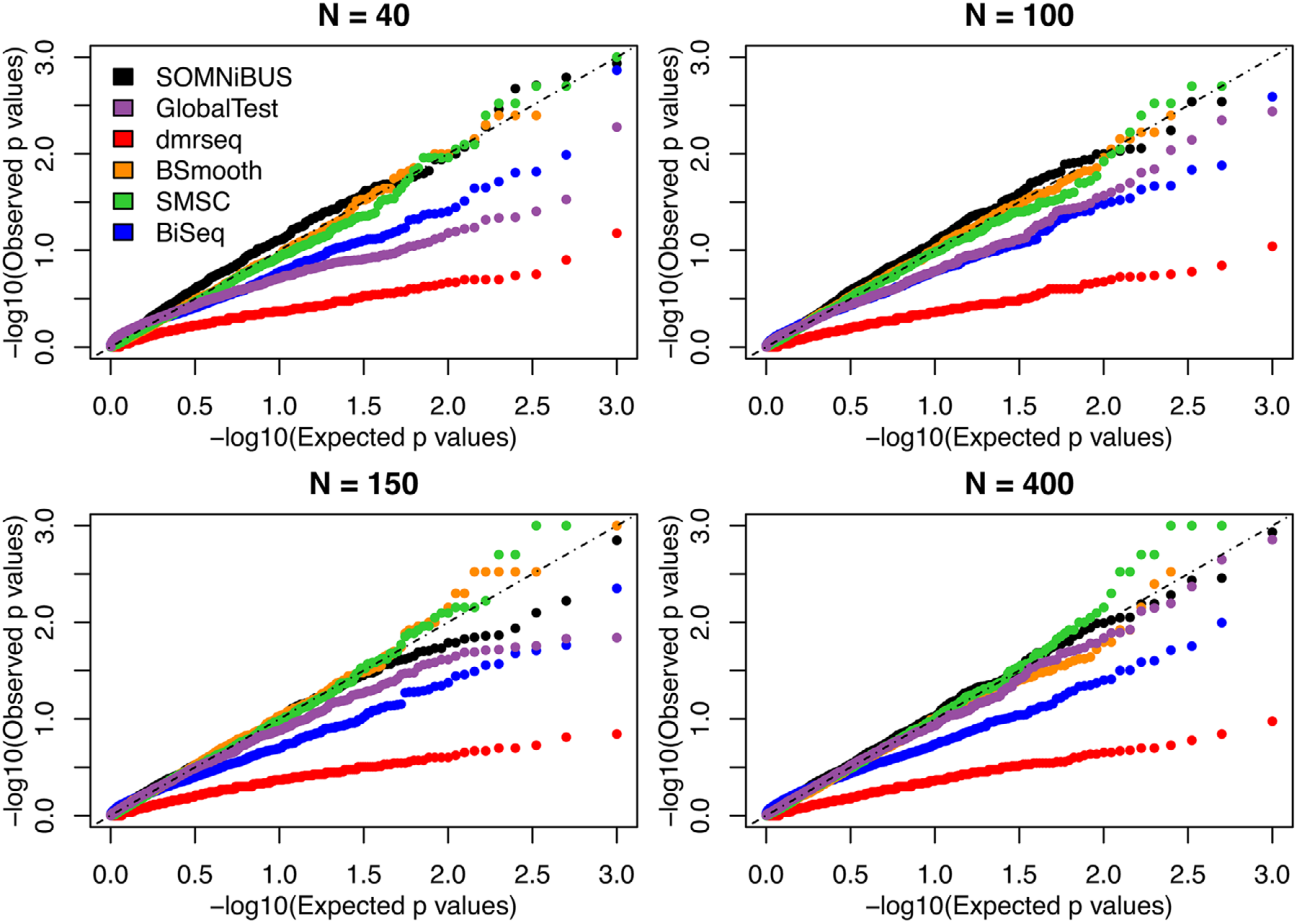
Quantile‐Quantile (Q‐Q) plots of the region‐based *P*‐values for the null covariate *Z*_3_, obtained from the six methods, over 1000 simulations. Data were generated without error with a range of sample sizes (N=40,100,150,400), under simulation Scenario 1. Here, the Expected *P*‐values are uniformly distributed numbers, equal to =(1/1001,2/1001,…,1000/1001).

**FIGURE 6:**
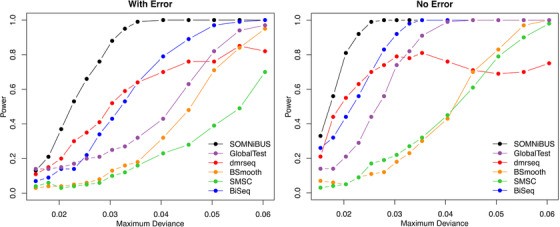
Powers to detect DMRs using the six methods for the 14 simulation settings in Scenario 2 under different levels of maximum deviation between $\pi_{0}\left(t\right)$ and $\pi_{1}\left(t\right)$, calculated over 100 simulations. (Sample size N=100).

Figures 5 and 6 further demonstrate the performance of the proposed regional test, described in Section 2.4.2. The results of type I error rate and power from our smoothed‐EM method are compared to the five existing methods GlobalTest, dmrseq, BSmooth, SMSC, and BiSeq. Figure 5 shows the distributions of *P*‐values for the regional effect of the null covariate *Z*_3_, obtained from the six methods. Because none of GlobalTest, dmrseq, BSmooth nor BiSeq accounts for the presence of experimental errors, for a fair comparison, the simulated data used in Figure 5 were generated without error (ie, $p_{0}=1-p_{1}=0$). The corresponding results for data generated with error are shown in the Supporting Information Figure S2. Figure 5 shows that the region‐based *P*‐values for *Z*_3_, calculated from our smoothed‐EM approach (black dots), are uniformly distributed, under all sample sizes considered. In contrast, the distributions of *P*‐values from dmrseq, BiSeq, and GlobalTest are biased away from what would be expected under the null. Because the inferences for BSmooth and SMSC are drawn from permutations, both methods are able to control type I error. Similar results were observed when data were generated with error. The results demonstrate that the distribution of the SOMNiBUS region‐based statistics under the null is well calibrated even at a relatively small sample size N=40, indicating the proposed regional zero effect test can correctly control the type I error. Figure 6 shows the powers of the six methods for detecting DMRs under the 14 settings of methylation patterns displayed in Figure 2. In Figure 6, the left panel presents the results obtained from data with error ($p_{0}=0.003$ and $1-p_{1}=0.1$); the right panel presents results obtained from data without error ($p_{0}=1-p_{1}=0$). Figure 6 shows that the proposed smoothed‐EM method has a higher power than the five alternative methods; this superiority is even more pronounced when the data were generated with error.

In summary, SOMNiBUS provides accurate estimates for smooth covariate effects; when compared with the existing methods considered here, SOMNiBUS exhibits greater power to detect DMRs, while correctly controlling type I error rates.

## 5 DISCUSSION

Currently, there are no tools for estimating smooth covariate effects for bisulfite sequencing data. In this paper, we propose and evaluate a method, SOMNiBUS, that aims to fill this gap. Our contribution is threefold. First, we develop a novel model to represent the bisulfite sequencing data from multiple samples, which naturally accounts for variable read depth, experimental errors, and a mixture of cell types. Second, we provide a formal inference for smooth covariate effects across a region of interest, where outcomes may be contaminated by errors. Third, we construct a region‐based statistic with a simple chi‐squared limiting distribution for jointly testing multiple coefficients in the presence of penalization. Results from simulations and one real data example show that the new method captures important underlying methylation patterns, provides accurate estimates of covariate effects, and correctly quantifies the underlying uncertainty in the estimates. The method has been implemented in R package SOMNiBUS, which will be submitted to CRAN.

Our method assumes that the error parameters *p*_0_ and *p*_1_ are known and do not vary across the region of interest. Although it is conceptually feasible to estimate these parameters by an EM‐type approach, the added computational burden in the E step would be substantial, because the complete‐data likelihood is not linear in the methylated counts. Moreover, there are cases in which these parameters can actually be measured, for example by adding spike‐in sequences of DNA that are known in advance to be methylated or unmethylated into the bisulfite sequencing procedure. The results from the sensitivity analyses (Supplementary Information Figures S3 and S4) show that misspecified error rates can introduce a minor bias in regional *P*‐values; however, this is not likely to affect the power of our tests, as demonstrated in the Supporting Information Table S2. An extension worth exploring in the future will be to accommodate variations of *p*_0_ and *p*_1_ across genomic positions into our model. For example, the error rates could be modeled to depend on prior annotation information, CG content, or on the experimental quality in the test region.

Another potential limitation of our inference procedures is the treatment of the smoothing parameters as fixed, disregarding the uncertainty in estimating them. However, our simulation results show that both the confidence interval coverage at each site and the type I error rates at the region level, are close to their nominal value; hence, our compromise does not lead to a major efficiency loss. Nevertheless, this uncertainty could be accounted for by adding in our method an approximate correction, as proposed by Kass and Steffey (1989), or considering a full Bayesian inference where one could specify a prior distribution for the smoothing parameters ***λ***.

There is a substantial computational burden in our estimation algorithm, because the M step includes two inner iteration schemes: P‐IRLS for updating smooth covariate effects, and Newton's optimization for updating smoothing parameters. A summary of runtimes for SOMNiBUS and the five alternative methods is displayed in the Supporting Information Figure S5. This figure shows that SOMNiBUS requires longer computational times than GlobalTest, BSmooth, SMSC and BiSeq, but less than dmrseq. Note that our proposed method, SOMNiBUS, is capable of estimating the effects of multiple covariates simultaneously, whereas, other methods require repeating the analysis for each covariate, which will multiply the runtimes. Our algorithm could be sped up by transforming the methylation proportions into a continuous‐type variable, as in Korthauer *et al*. (2019), which allows us to replace the P‐IRLS with the ordinary least square, and mitigate any instability in estimation of methylation levels near the boundaries (proportions of zero or one). However, transforming the count outcome into a continuous variable causes extra difficulties in the Expectation step, for which no closed‐form exact expression is available.

The proposed approach is tailored to targeted bisulfite sequencing data. Another future direction is to extend our method to WGBS data. This requires first partitioning whole genome into regions or using a sliding window; optimal partitioning or choices of window sizes are challenges to be met. We recommend for the moment that algorithms such as BSmooth or dmrseq be used to find interesting regions. These regions could then be re‐analyzed with SOMNiBUS to more comprehensively and simultaneously estimate covariate influences on methylation.

6

## Supporting information

Web Appendices, Tables, and Figures, referenced in Section 2, 3, 4 and 5, are available with this paper at the Biometrics website on Wiley Online Library. Codes to replicate the simulation results in the article are deposited in the Github repository https://github.com/kaiqiong/SOMNiBUS_Simu. The R package, SOMNiBUS, implementing the proposed method is available from Github at https://github.com/GreenwoodLab/SOMNiBUS, with a user guide.

## Data Availability

The data that support the findings in this paper are available on request from the co‐author Dr. Marie Hudson. The data are not publicly available due to privacy or ethical restrictions.
