# Supplementary material for: A novel statistical method for modeling covariate effects in bisulfite sequencing derived measures of DNA methylation
Source: Biometrics. 2020 Jun 5;77(2):424–38. doi: 10.1111/biom.13307 (PMC8359306; doi:10.1111/biom.13307)
Supplement: Supplementary file 1 — Web Appendices, Tables, and Figures, referenced in Section 2, 3, 4 and 5, are available with this paper at the Biometrics website on Wiley Online Library. Codes to replicate the simulation results in the article are deposited in the Github repository https://github.com/kaiqiong/SOMNiBUS_Simu. The R package, SOMNiBUS, implementing the proposed method is available from Github at https://github.com/GreenwoodLab/SOMNiBUS, with a user guide. [file BIOM-77-424-s001.pdf]

Supporting Information for "A novel statistical method for modeling covariate effects in bisulfite sequencing derived measures of DNA methylation" by

Kaiqiong Zhao, Karim Oualkacha, Lajmi Lakhal-Chaieb, Aurélie Labbe, Kathleen Klein, Antonio Ciampi, Marie Hudson, Inés Colmegna, Tomi Pastinen, Tieyuan Zhang, Denise Daley, Celia M.T. Greenwood

May 13, 2020

This Supporting Information includes detailed derivations, proofs, additional simulation and data application results, and software and data guidance.

1 APPENDIX

1.1 Appendix A: the form of the spanned design matrix

The design matrix $\underset{[M\times K]}{\mathbb{X}} = \left(\boldsymbol{B}^{(Z_0)}, \boldsymbol{B}^{(Z_1)}, \dots \boldsymbol{B}^{(Z_P)}\right)$ consists of the blocks

$$\underset{(M\times L_p)}{\boldsymbol{B}^{(Z_p)}} = \begin{pmatrix} B_1^{(p)}(t_{11}) \times Z_{p1} & \dots & B_{L_p}^{(p)}(t_{11}) \times Z_{p1} \\ \vdots & & \vdots \\ B_1^{(p)}(t_{1m_1}) \times Z_{p1} & \dots & B_{L_p}^{(p)}(t_{1m1}) \times Z_{p1} \\ B_1^{(p)}(t_{21}) \times Z_{p2} & \dots & B_{L_p}^{(p)}(t_{21}) \times Z_{p2} \\ \vdots & & \vdots \\ B_1^{(p)}(t_{2m_2}) \times Z_{p2} & \dots & B_{L_p}^{(p)}(t_{2m2}) \times Z_{p2} \\ \vdots & & \vdots \\ B_1^{(p)}(t_{Nm_N}) \times Z_{pN} & \dots & B_{L_p}^{(p)}(t_{NmN}) \times Z_{pN} \end{pmatrix} \quad \text{for } p = 0, 1, \dots P,$$

where $Z_{0i} \equiv 1$ for $i = 1, 2 \dots N$.

1.2 Appendix B: the P-IRLS step given the values of smoothing parameters

One update in the P-IRLS estimation from step r to step $r+1$ is

$$\boldsymbol{\alpha}^{(r+1)} = (\mathbb{X}^T \boldsymbol{W}^{(r)} \mathbb{X} + \boldsymbol{A}_{\boldsymbol{\lambda}})^{-1} \mathbb{X}^T \boldsymbol{W}^{(r)} \widetilde{\boldsymbol{S}}^{(r)},$$

where $\boldsymbol{W}^{(r)} = \text{Diag}\{w_{11}, \dots w_{1m_1}, w_{21}, \dots w_{2m_2}, \dots w_{Nm_N}\} \in \mathcal{R}^{M\times M}$ with $w_{ij} = \pi_{ij}^{(r)}(1 - \pi_{ij}^{(r)})$ is the weight matrix, and $\widetilde{\boldsymbol{S}}^{(r)} = \left(\widetilde{S}_{11}^{(r)}, \dots \widetilde{S}_{1m_1}^{(r)}, \widetilde{S}_{21}^{(r)}, \dots \widetilde{S}_{2m_2}^{(r)}, \dots \widetilde{S}_{Nm_N}^{(r)}\right) \in \mathcal{R}^M$ with $\widetilde{S}_{ij}^{(r)} = g\left(\pi_{ij}^{(r)}\right) + g'\left(\pi_{ij}^{(r)}\right) \quad \left(\eta_{ij}^{\star} - \pi_{ij}^{(r)}\right)$ is the vector of adjusted response (also called pseudo response) variables.

1.3 Appendix C: Laplace approximated restrictive log-likelihood

In the outer optimization, $\boldsymbol{\lambda}$ is estimated by maximizing the Laplace approximated restricted likelihood (Wood, 2011), denoted by $l_r(\boldsymbol{\lambda})$,

$$2l_r(\boldsymbol{\lambda}) = 2l(\widehat{\boldsymbol{\alpha}_{\boldsymbol{\lambda}}}) + \log(|\boldsymbol{A}_{\boldsymbol{\lambda}}|) - \widehat{\boldsymbol{\alpha}_{\boldsymbol{\lambda}}}^{\boldsymbol{T}} \boldsymbol{A}_{\boldsymbol{\lambda}} \widehat{\boldsymbol{\alpha}_{\boldsymbol{\lambda}}} - \log(|\boldsymbol{H} + \boldsymbol{A}_{\boldsymbol{\lambda}}|) + M_A log(2\pi)$$

with $\boldsymbol{H} = -\boldsymbol{\partial^2 l(\alpha)/\partial\alpha\partial\alpha^T} = \mathbb{X}^T \boldsymbol{\Lambda_X W} \mathbb{X}$. Here, $l(\alpha)$ is the log-likelihood derived from the binomial distribution as defined in the main manuscript, and $\boldsymbol{\Lambda_X} = \text{Diag}\{X_{11}, \ldots, X_{1m_1}, X_{21}, \ldots, X_{2m_2}, \ldots, X_{Nm_N}\}$ is the diagonal matrix with values of read-depths. $\boldsymbol{H}$ depends on the vector $\boldsymbol{\lambda}$ via the dependence of $\boldsymbol{A}_{\boldsymbol{\lambda}}$ and $\widehat{\boldsymbol{\alpha}}$ on $\boldsymbol{\alpha}$, and M_A is the dimension of the null space of $\boldsymbol{A}_{\boldsymbol{\lambda}}$.

1.4 Appendix D: Proof of Theorem 1

The proof of Theorem 1 is based on Lemmas 1 and 2. Lemma 1 shows the second derivatives of the conditional log-likelihood $Q(\boldsymbol{\alpha} \mid \boldsymbol{\alpha}^\star)$, and Lemma 2 obtains the Hessian matrix of the marginal log-likelihood of $\boldsymbol{Y}$.

Lemma 1. *The second derivative of the conditional log-likelihood function $Q(\boldsymbol{\alpha} \mid \boldsymbol{\alpha}^\star)$ with respect to $\boldsymbol{\alpha}$ is*

$$\frac{\partial^2 Q(\boldsymbol{\alpha} \mid \boldsymbol{\alpha}^\star)}{\partial \boldsymbol{\alpha}\, \partial \boldsymbol{\alpha}^{\boldsymbol{T}}} = -\mathbb{X}^T \boldsymbol{\Lambda_X W} \mathbb{X} - \boldsymbol{A}_{\boldsymbol{\lambda}}, \tag{1}$$

where $\boldsymbol{W} = Diag\{w_{11}, \ldots w_{1m_1}, w_{21}, \ldots, w_{2m_2}, \ldots w_{Nm_N}\} \in \mathcal{R}^{M\times M}$ is the weight matrix with element $w_{ij} = \pi_{ij}(1-\pi_{ij})$, and $\boldsymbol{\Lambda_X} = Diag\{X_{11}, \ldots, X_{1m_1}, X_{21}, \ldots, X_{2m_2}, \ldots, X_{Nm_N}\}$ is the diagonal matrix with values of read-depths. The mixed second derivatives of $Q(\alpha \mid \alpha^\star)$ with respect to $\boldsymbol{\alpha}$ and $\boldsymbol{\alpha}^\star$ are

$$\frac{\partial^2 Q(\boldsymbol{\alpha} \mid \boldsymbol{\alpha}^\star)}{\partial \boldsymbol{\alpha}\, \partial \boldsymbol{\alpha}^{\star \boldsymbol{T}}} = \mathbb{X}^T \boldsymbol{\Lambda}_{\boldsymbol{\eta}}^\star \boldsymbol{W}^\star \mathbb{X} \tag{2}$$

where $\boldsymbol{W}^{\star}$ *is the weight matrix evaluated at* $\pi^{\star}$*, which is the current iteration estimates and* $\boldsymbol{\Lambda}_{\boldsymbol{\eta}}^{\star}$ *is a diagonal matrix with diagonal elements* $\delta_{ij}^{\star}$*, defined as,*

$$\delta_{ij}^{\star} = \frac{Y_{ij} p_1 p_0}{\left[p_1 \pi_{ij}^{\star} + p_0 (1 - \pi_{ij}^{\star})\right]^2} + \frac{(X_{ij} - Y_{ij})(1 - p_1)(1 - p_0)}{\left((1 - p_1)\pi_{ij}^{\star} + (1 - p_0)(1 - \pi_{ij}^{\star})\right)^2}. \tag{3}$$

Proof. The Q function takes the form

$$Q(\boldsymbol{\alpha} \mid \boldsymbol{\alpha}^{\star}) = \sum_{i=1}^{N} \sum_{j=1}^{m_i} \left\{ \eta_{ij}^{\star} \theta_{ij} - X_{ij} \log(1 + e^{\theta_{ij}}) \right\} - \frac{1}{2} \boldsymbol{\alpha}^{\boldsymbol{T}} \boldsymbol{A}_{\boldsymbol{\lambda}} \boldsymbol{\alpha},$$

where $\theta_{ij} = \log\left(\pi_{ij} / (1 - \pi_{ij})\right)$. The first term is the binomial log-likelihood function evaluated at $\eta^{\star}(\boldsymbol{\alpha}^{\star})$, the conditional expectations of the true outcome S_{ij}.

We derive the first and second derivatives of $Q(\boldsymbol{\alpha} \mid \boldsymbol{\alpha}^{\star})$ with respect to $\boldsymbol{\alpha}$ and $\boldsymbol{\alpha}^{\star}$. First, it is easy to show that

$$\frac{\partial Q(\boldsymbol{\alpha} \mid \boldsymbol{\alpha}^{\star})}{\partial \boldsymbol{\alpha}} = \sum_{(i,j)} \left\{ \left[\eta_{ij}^{\star} - X_{ij} \pi_{ij}\right] \left[(\mathbb{X})_{(l,\cdot)}\right]^{T} \right\} - \begin{Bmatrix} \lambda_0 \boldsymbol{A_0} \boldsymbol{\alpha_0} \\ \lambda_1 \boldsymbol{A_1} \boldsymbol{\alpha_1} \\ \cdots \\ \lambda_P \boldsymbol{A_P} \boldsymbol{\alpha_P} \end{Bmatrix}. \tag{4}$$

Here we use $(\mathbb{X})_{(l,\cdot)}$ to denote the lth row of the design matrix, which is the row corresponding to the CpG j of sample i.

Differentiation of equation (4) with respect to $\boldsymbol{\alpha}$ and $\boldsymbol{\alpha}^{\star}$ yields respectively

$$\left(\frac{\partial^2 Q(\boldsymbol{\alpha} \mid \boldsymbol{\alpha}^{\star})}{\partial \boldsymbol{\alpha}\, \partial \boldsymbol{\alpha}^{\boldsymbol{T}}}\right)_{(m,m')} = \sum_{(i,j)} \left\{ -X_{ij} \pi_{ij} (1 - \pi_{ij}) (\mathbb{X})_{(l,m)} (\mathbb{X})_{(l,m')} \right\} - \lambda_{\widetilde{p}} (\boldsymbol{A_p})_{(k,\widetilde{k})} \mathcal{I}_{(m,m')}, \tag{5}$$

$$\left(\frac{\partial^2 Q(\boldsymbol{\alpha} \mid \boldsymbol{\alpha}^{\star})}{\partial \boldsymbol{\alpha}\, \partial \boldsymbol{\alpha}^{\star \boldsymbol{T}}}\right)_{(m,m')} = \sum_{(i,j)} \left\{ \frac{\partial \eta_{ij}^{\star}}{\partial \pi_{ij}^{\star}} \pi_{ij}^{\star} (1 - \pi_{ij}^{\star}) (\mathbb{X})_{(l,m)} (\mathbb{X})_{(l,m')} \right\}, \tag{6}$$

for $m, m' = 1, 2, \ldots K$. In the above formulas, $(\bullet)_{(m,m')}$ represents the (m, m') entry of a matrix. $\mathcal{I}_{(m,m')} = 1$ if α_m and $\alpha_{m'}$ are the basis coefficients for the same functional parameter $\beta_p(t)$, and $\mathcal{I}_{(m,m')} = 0$ otherwise. For the pairs (m, m') that satisfy $\mathcal{I}_{(m,m')} = 1$,

we use k and $\widetilde{k}$ to denote the index of the bases associated with coefficients α_m and $\alpha_{m'}$; in other words, α_m and $\alpha_{m'}$ are the kth and $\widetilde{k}^{\text{th}}$ basis coefficients in the linear expansion that are used to represent functional parameter $\beta_p(t)$. In addition, the $\partial\eta^\star_{ij}/\partial\pi^\star_{ij}$ in the formula (6) equals to $\delta^\star_{ij}$, as defined in (3). The values of δ_{ij} reduce to 0 when error parameters $p_0 = 1 - p_1 = 0$.

Finally, we rewrite the expressions in (5) and (6) in a compact way using matrices $\boldsymbol{\Lambda_X}, \boldsymbol{W}, \boldsymbol{\Lambda^\star_\eta}$, and obtain the expressions in (1) and (2). □

Lemma 2. *The Hessian matrix of the marginal log-likelihood of Y has the form*

$$\mathcal{H}(\boldsymbol{\alpha}) = \mathbb{X}^T(-\boldsymbol{\Lambda}_X + \boldsymbol{\Lambda}_\eta)\boldsymbol{W}\mathbb{X} - \boldsymbol{A_\lambda},$$

where $\boldsymbol{\Lambda}_\eta$ is a diagonal matrix with elements δ_{ij}, which is of the similar form as $\delta^\star_{ij}$ in (3) but replacing $\pi^\star_{ij}$ with π_{ij}.

Proof. Due to the presence of the latent methylation state S_{ij}, the observed counts Y_{ij} follow a mixture of two binomial distributions. A direct calculation of the observed Fisher information (Hessian matrix) from this marginal distribution is analytically intractable. However, Oakes (1999) showed that, although the marginal log-likelihood itself is not expressible, its observed Fisher information, can be expressed in terms of the Q function (i.e. the conditional expectation of the log-likelihood of S_{ij} given the observed data Y_{ij}) and its derivatives. Specifically, we rely on the work done by Oakes (1999) and calculate the Hessian matrix of the marginal log-likelihood of Y for parameter $\boldsymbol{\alpha}$, $\mathcal{H}(\boldsymbol{\alpha})$, as the sum of two second derivatives of the Q function,

$$\mathcal{H}(\boldsymbol{\alpha}) = \left\{ \frac{\partial^2 Q(\boldsymbol{\alpha} \mid \boldsymbol{\alpha}^\star)}{\partial\boldsymbol{\alpha}\, \partial\boldsymbol{\alpha^T}} + \frac{\partial^2 Q(\boldsymbol{\alpha} \mid \boldsymbol{\alpha}^\star)}{\partial\boldsymbol{\alpha}\, \partial\boldsymbol{\alpha^{\star T}}} \right\}\Bigg|_{\boldsymbol{\alpha}^\star=\boldsymbol{\alpha}}.$$

Using the results in Lemma 1, it can be easily shown that the Hessian matrix $\mathcal{H}(\boldsymbol{\alpha})$ of the marginal log-likelihood of Y is

$$\mathcal{H}(\boldsymbol{\alpha}) = \mathbb{X}^T(-\boldsymbol{\Lambda}_X + \boldsymbol{\Lambda}_\eta)\boldsymbol{W}\mathbb{X} - \boldsymbol{A_\lambda}.$$

The diagonal matrix $\mathbf{\Lambda}_{\boldsymbol{\eta}}$ will be equal to 0 when error parameters $p_0 = 1 - p_1 = 0$, which corresponds to the case with no experimental error present in the data. □

Theorem 1. *Under the usual regularity conditions for maximum likelihood, we have the following asymptotic results for the estimators $\widehat{\boldsymbol{\alpha}}$ obtained from the smoothed-EM algorithm,*

$$\sqrt{M}\,(\widehat{\boldsymbol{\alpha}} - \boldsymbol{\alpha}) \xrightarrow{\mathcal{L}} \boldsymbol{MVN}_K(\mathbf{0}, \boldsymbol{\mathcal{I}}^{-1}), \quad \text{as } M \to \infty.$$

Here, K is the dimension of the spline coefficients $\boldsymbol{\alpha}$, and $\boldsymbol{\mathcal{I}} = \mathbb{E}\left[-\mathcal{H}_{ij}(\boldsymbol{\alpha})\right]$. Specifically $\mathcal{H}_{ij}(\boldsymbol{\alpha})$ has the form

$$\mathcal{H}_{ij}(\boldsymbol{\alpha}) = \mathbb{X}_{(l,)}^T \left(-X_{ij}w_{ij} + \delta_{ij}w_{ij}\right) \mathbb{X}_{(l,)} - \boldsymbol{A}_{\boldsymbol{\lambda}}, \tag{7}$$

where $\mathbb{X}_{(l,)}$ is the lth row of the design matrix $\mathbb{X}$, which corresponds to the CpG j of sample i, and $w_{ij} = \pi_{ij}(1 - \pi_{ij})$ is the element of the weight matrix.

Proof. Based on the results in Lemma 2, we can show that the Hessian matrix calculated from the individual contribution from observation i at position j, $\mathcal{H}_{ij}(\boldsymbol{\alpha})$, can be expressed as in equation (7).

Hence, the asymptotic result follows from the fact that smoothed-EM estimate $\widehat{\boldsymbol{\alpha}}$ is a MLE of $\boldsymbol{\alpha}$ for the marginal distribution of $\boldsymbol{Y}$ (Dempster et al., 1977), and $\mathcal{H}_{ij}(\boldsymbol{\alpha})$ is the Hessian matrix of $\boldsymbol{\alpha}$ for the marginal distribution of $\boldsymbol{Y}_{ij}$ (Oakes, 1999).

□

2 ADDITIONAL SIMULATION RESULTS

In this section, we present additional Figures and Tables referenced in Sections 4 and 5 in the main manuscript.

2.1 Simulation settings and additional results for Type I Error assessment

Figure S1 displays the 14 simulation settings of functional parameters $\beta_0(t)$ and $\beta_1(t)$ in Scenario 2. Each pairs of $\beta_0(t)$ and $\beta_1(t)$ correspond to the 14 settings for $\pi_0(t)$ and $\pi_1(t)$ shown in Figure 2 in the main manuscript (the black solid lines). Once we fixed the shapes of $\pi_0(t)$ and $\pi_1(t)$ (in Figure 2 in the main manuscript), $\beta_0(t)$ and $\beta_1(t)$ have the forms

$$\begin{aligned} \beta_0(t) &= \log \frac{\pi_0(t)}{1-\pi_0(t)} \\ \beta_1(t) &= \log \frac{\pi_1(t)}{1-\pi_1(t)} - \beta_0(t). \end{aligned}$$

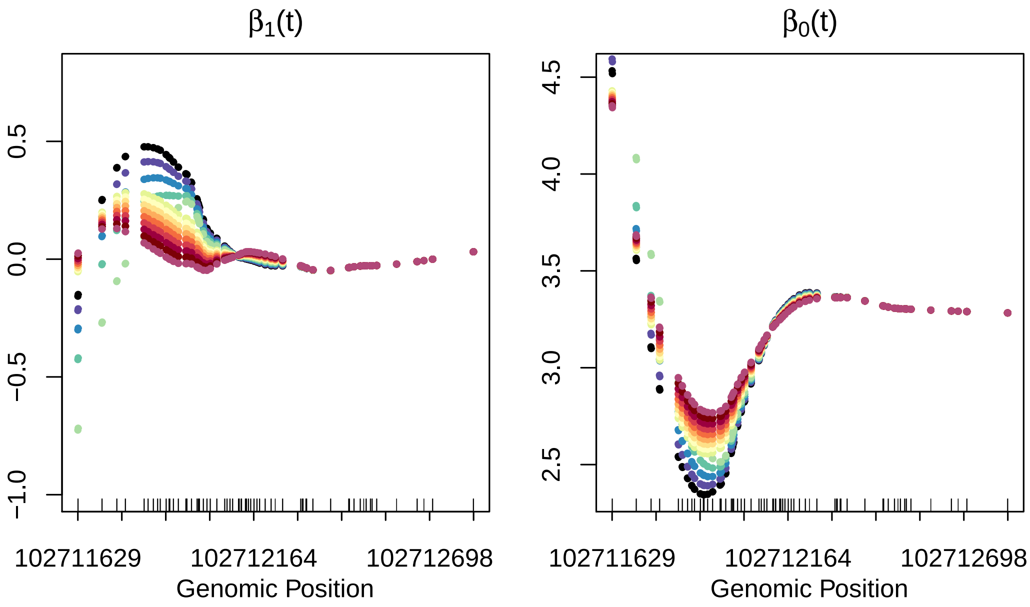

Figure S1: The 14 simulation settings of functional parameters $\beta_0(t)$ and $\beta_1(t)$ in Scenario 2, which correspond to the 14 settings for $\pi_0(t)$ shown in Figure 2 in the main manuscript.

Table S1: Simulation settings outlined in Section 4.1 in the main manuscript, for the functional parameters $\beta_p(t)$, sample size N, and error parameters p_0 and p_1.

Simulation parameters	Possibilities
$\beta_p(t)$	Scenario 1: three covariates: $Z_1 \sim Bernoulli(0.51)$, $Z_2 \sim Bernoulli(0.58)$ and $Z_3 \sim Bernoulli(0.5)$ with effects $\beta_1(t), \beta_2(t)$ and $\beta_3(t)$ and intercept $\beta_0(t)$, shown in the red curves in Figure 1 of the main manuscript.
	Scenario 2: one covariate $Z \sim Bernoulli(0.5)$ with 14 different settings of $(\beta_0(t), \beta_1(t))$, as shown in Figure S1 in the Supporting Information.
N	(40, 100, 150, 400)
(p_0, p_1)	$p_0 = 0.003; p_1 = 0.9$

Table S1 summarizes the simulation settings outlined in Section 4.1 in the main manuscript. Figure S2 shows the distribution of p-values for the regional effect of the null covariate Z_3 when data were generated with error.

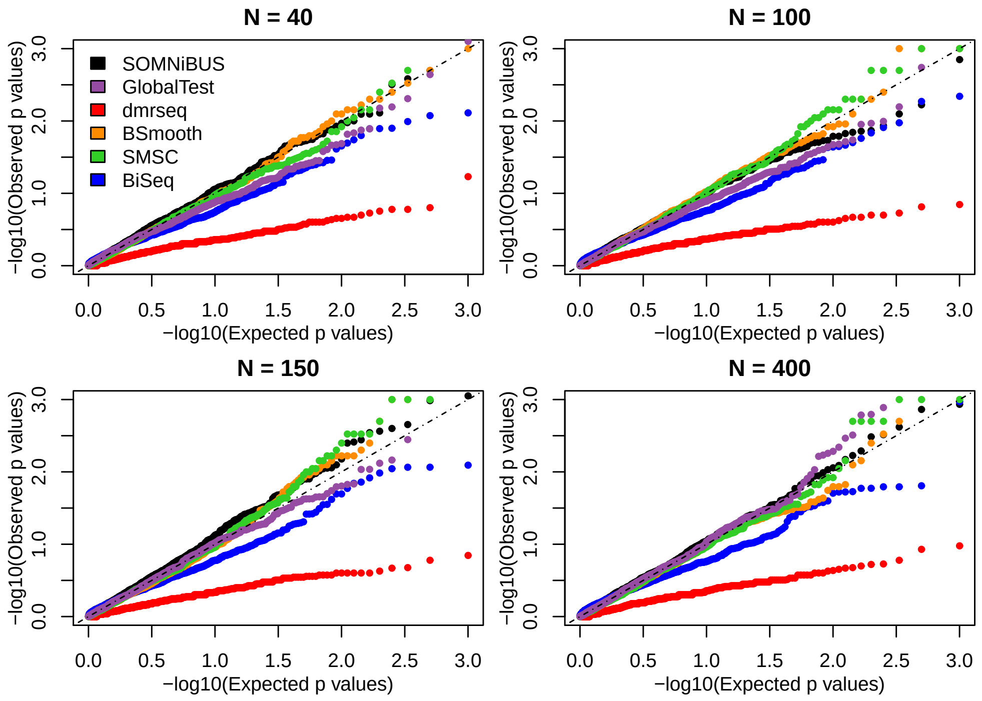

Figure S2: Quantile-Quantile (Q-Q) plots of the region-based p-values for the null covariate Z_3, obtained from the six methods, over 1000 simulations. Data were generated **with error** with a range of sample sizes ($N = 40, 100, 150, 400$), under simulation Scenario 1. Here, the Expected p-values are uniformly distributed numbers, equal to $= (1/1001, 2/1001, \ldots, 1000/1001)$ and both axes are transformed with $-log10(p)$.

2.2 Sensitivity to Bisulfite Sequencing Error Parameters

We explored additional simulation scenarios where the error parameters p_0 and p_1 were misspecified. Specifically, the data were generated subject to errors $p_0 = 0.003$ and $1 - p_1 = 0.1$ but analyses were conducted using a grid of values for p_0 and p_1, constructed from $p_0 = (0, 0.003, 0.005, 0.1, 0.2)$ and $p_1 = (0.88, 0.89, 0.9, 0.95, 1)$. We considered the 14 settings of Scenario 2 that were described in Section 4.1 and graphed in Figure 2 in the main manuscript. These results are shown in columns named S1-S14 in Table S2. We also

included one simulation with a null covariate effect and with varying error parameters, and these results are shown in a column named S0 in Table S2. These 15 settings S0-S14 correspond to increasing levels of differences between methylation patterns from two groups, i.e. with increasing maximum deviation (MD) between the methylation levels of $Z = 0$ and $Z = 1$.

The powers to detect DMRs for different configurations of p_0 and p_1 under each simulation setting (S0-S14) were given in Table S2 (note that the power under S0 is the type I error rate). The actual region-based p-values from the 100 simulations for setting S1 with small methylation differences, and setting S14 with large methylation differences, were displayed in Figure S3 and Figure S4, respectively. In Figures S3 and S4, the region-based p-values using the (mis)specified p_0 and p_1 (vertical axis) were plotted against the ones using the correct p_0 and p_1 (horizontal axis).

Figure S3 and Figure S4 show that misspecified error rates can lead to minor differences in regional p-values from the ones with correctly-specified error rates. This difference tends to be greater when the effect size of the covariate of interest is large and when the bias in the error parameters are big. Despite the differences in the actual regional p-values, the powers under various misspecified error rates are shown to be similar to the case with known error rates, as demonstrated in Table S2. In addition, when the error rates are specified with strong bias, the EM algorithm will not converge. For example, for the simulation scenarios considered in Table S2, the analyses using $p_1 \leq 0.88$ failed to converge. This also provides a sign of error misspecification.

Table S2: Powers to detect DMRs using SOMNiBUS when the error parameters p_0 and p_1 were specified differently, under the 14 settings as shown in Figure 2 in the main manuscript (S1-S14) and 1 setting under Null (S0). The powers were calculated over 100 simulations and the data were generated based on the error parameters $p_0 = 0.003$ and $p_1 = 0.9$ (in gray shade), and sample size $N = 100$.

p_1	p_0	S0	S1	S2	S3	S4	S5	S6	S7	S8	S9	S10	S11	S12	S13	S14
	0	0.04	0.13	0.24	0.38	0.59	0.71	0.85	0.94	0.97	0.99	1	1	1	1	1
	0.003	0.04	0.13	0.24	0.38	0.59	0.71	0.85	0.94	0.97	0.99	1	1	1	1	1
0.88	0.005	0.04	0.13	0.24	0.38	0.59	0.71	0.85	0.94	0.97	0.99	1	1	1	1	1
	0.1	0.04	0.13	0.24	0.38	0.59	0.7	0.85	0.95	0.98	1	1	1	1	1	1
	0.2	0.04	0.13	0.24	0.38	0.58	0.7	0.85	0.95	0.98	1	1	1	1	1	1
	0	0.04	0.12	0.21	0.39	0.55	0.67	0.81	0.9	0.96	0.98	1	1	1	1	1
	0.003	0.04	0.12	0.21	0.39	0.55	0.67	0.81	0.9	0.96	0.98	1	1	1	1	1
0.89	0.005	0.04	0.12	0.21	0.39	0.55	0.67	0.81	0.9	0.96	0.98	1	1	1	1	1
	0.1	0.04	0.12	0.21	0.39	0.55	0.67	0.81	0.9	0.96	0.98	1	1	1	1	1
	0.2	0.04	0.12	0.21	0.38	0.55	0.67	0.81	0.9	0.96	0.98	1	1	1	1	1
	0	0.04	0.14	0.22	0.37	0.53	0.65	0.77	0.87	0.94	0.99	1	1	1	1	1
	0.003	0.04	0.14	0.22	0.37	0.53	0.65	0.77	0.87	0.94	0.99	1	1	1	1	1
0.9	0.005	0.04	0.14	0.22	0.37	0.53	0.65	0.77	0.87	0.94	0.99	1	1	1	1	1
	0.1	0.04	0.14	0.23	0.37	0.53	0.65	0.77	0.87	0.94	0.99	1	1	1	1	1
	0.2	0.04	0.14	0.23	0.37	0.52	0.65	0.77	0.87	0.93	0.99	1	1	1	1	1
	0	0.06	0.12	0.2	0.31	0.44	0.58	0.7	0.78	0.87	0.94	0.97	1	1	1	1
	0.003	0.06	0.12	0.2	0.31	0.43	0.58	0.7	0.78	0.87	0.94	0.97	1	1	1	1
0.95	0.005	0.06	0.12	0.2	0.31	0.43	0.58	0.7	0.78	0.87	0.94	0.97	1	1	1	1
	0.1	0.06	0.12	0.2	0.3	0.44	0.59	0.68	0.77	0.88	0.95	0.98	1	1	1	1
	0.2	0.06	0.11	0.2	0.32	0.43	0.59	0.68	0.78	0.87	0.95	0.99	1	1	1	1
	0	0.06	0.13	0.18	0.29	0.42	0.54	0.67	0.75	0.87	0.91	0.97	1	1	1	1
	0.003	0.06	0.13	0.18	0.29	0.42	0.54	0.67	0.75	0.87	0.91	0.97	1	1	1	1
1	0.005	0.06	0.13	0.18	0.29	0.42	0.54	0.67	0.75	0.87	0.91	0.97	1	1	1	1
	0.1	0.06	0.13	0.18	0.29	0.42	0.54	0.65	0.75	0.87	0.91	0.98	1	1	1	1
	0.2	0.06	0.13	0.18	0.27	0.42	0.52	0.66	0.75	0.86	0.91	0.97	1	1	1	1

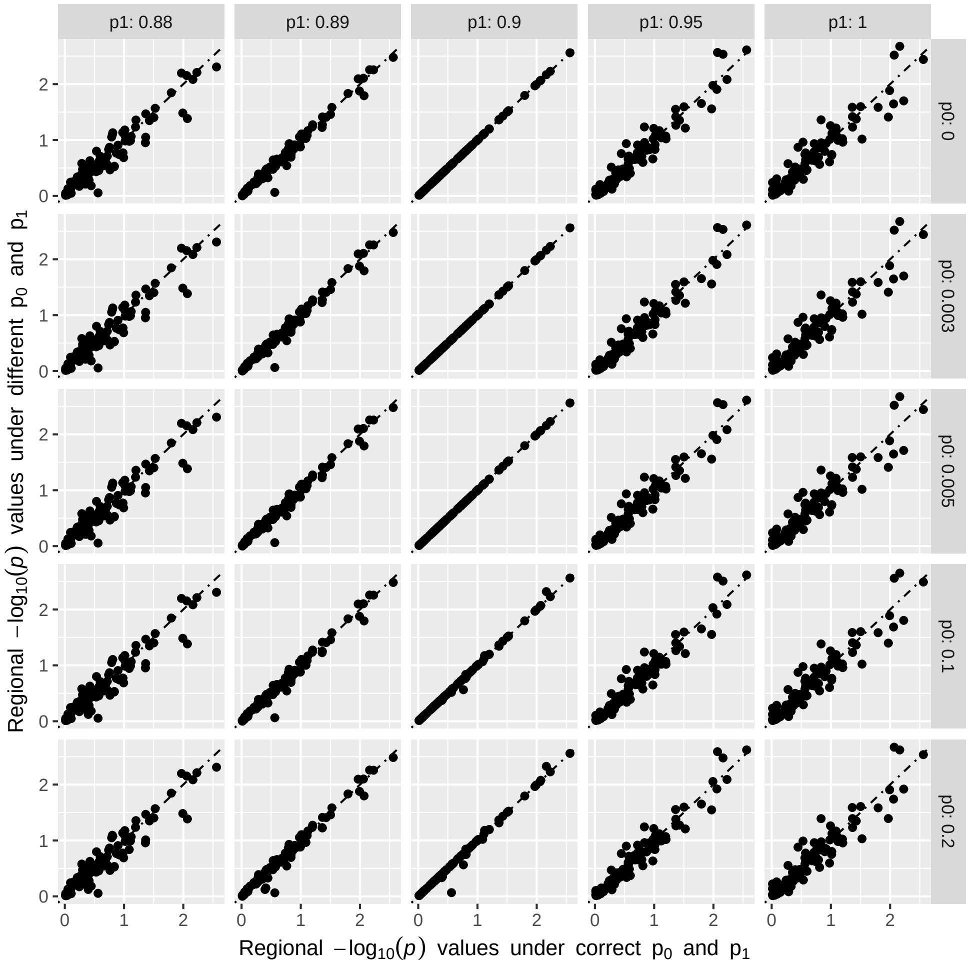

Figure S3: Scatter plots of the region-based p-values using the specified p_0 and p_1 (vertical axis) compared to the region-based p-values using the correct p_0 and p_1 (horizontal axis), for various settings of p_0 and p_1 specified in the facet labels, under 100 simulations. Here, data were simulated under S1 where MD between the methylation curves in two groups is 0.01 – small effect size.

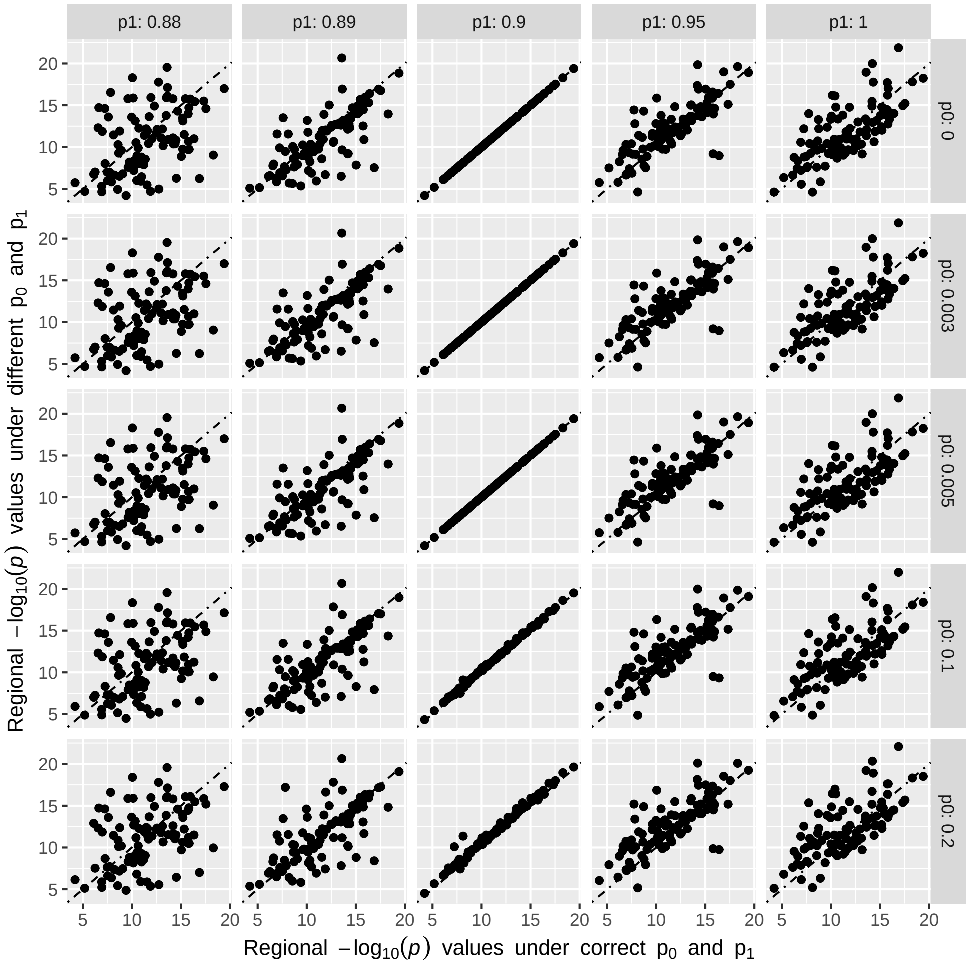

Figure S4: Scatter plots of the region-based p-values using the specified p_0 and p_1 (vertical axis) compared to the region-based p-values using the correct p_0 and p_1 (horizontal axis), for various settings of p_0 and p_1 specified in the facet labels, under 100 simulations. Here, data were simulated under S14 where MD between the methylation curves in two groups is 0.06 - large effect size.

2.3 Runtime Comparison

Figure S5 shows the runtime when fitting a single covariate using the methods under investigation. For dmrseq, we used three different numbers of permutations for comparison

(10, 100 and 500). SOMNiBUS No Error refers to assuming no sequencing errors in SOMNiBUS, which reduces the full model to a pure generalized additive model. Figure S5 shows that `SOMNiBUS` requires longer computational times than `GlobalTest`, `BSmooth`, `SMSC` and `BiSeq`, but less than `dmrseq`. Note that our proposed method, `SOMNiBUS`, is capable of estimating the effects of multiple covariates simultaneously, whereas, other methods require repeating the analysis for each covariate, which will multiply the runtime by the number of covariates.

3 ADDITIONAL DATA APPLICATION RESULTS

In addition to the *BANK1* region (Orozco et al., 2009), described in Section 3 in the main manuscript, we considered three more regions which overlap with genes *BLK*, *HLA-DRB* and *PTPN22*. These genes have been known associated with risk of rheumatoid arthritis (RA) (Zhang et al., 2012; Balsa et al., 2010; Hinks et al., 2006). We applied our method `SOMNiBUS`, along with the five alternative methods—`BiSeq`, `BSmooth`, `SMSC`, `dmrseq` and `GlobalTest`—to each targeted region of interest. Table S3 presents the region-based p-values for covariate effects on the four methylation regions. This table shows that `SOMNiBUS` reports smaller regional p-values, and exhibits an improved power to detect these RA-related methylation regions, as compared to the alternative methods.

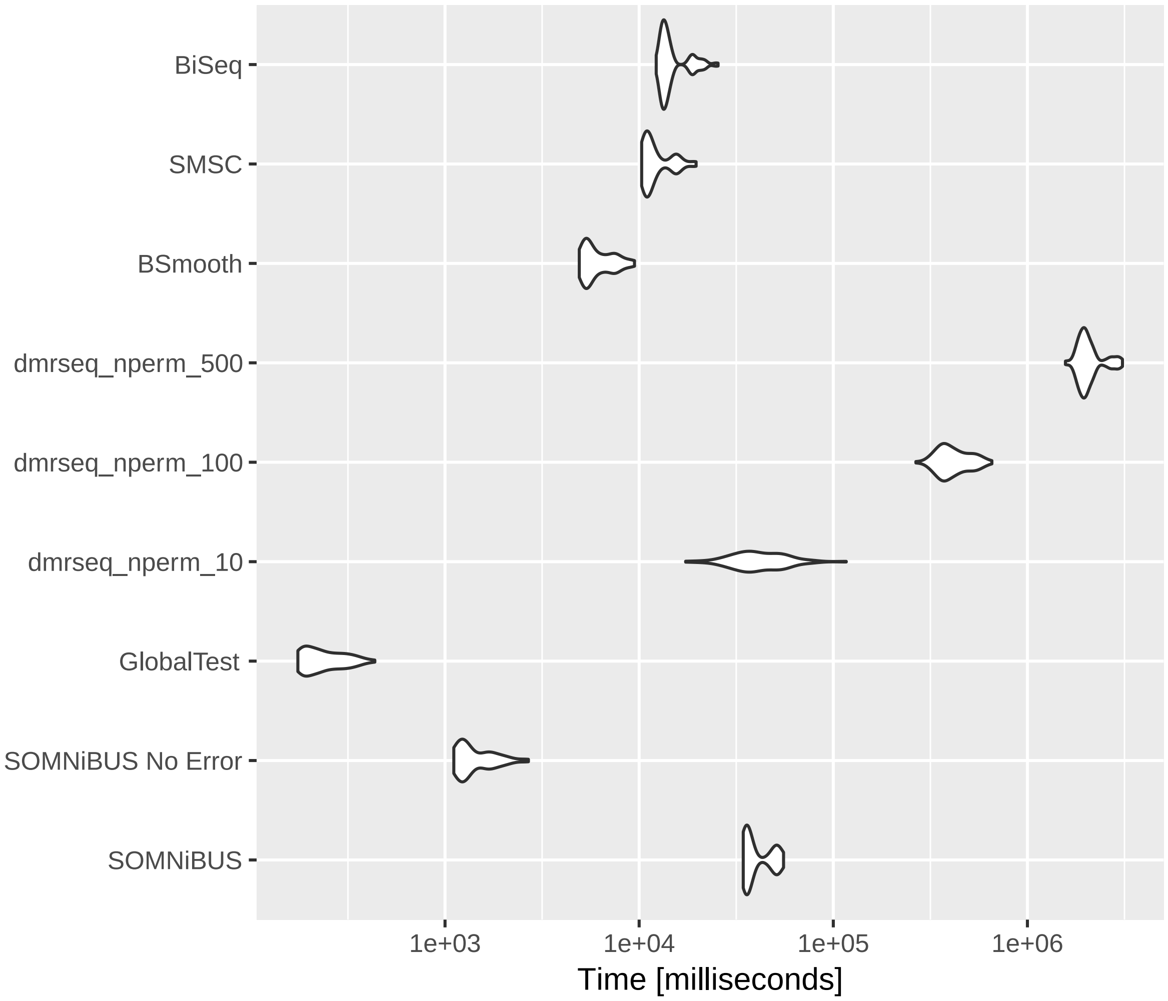

Figure S5: Summary of runtime under 100 replications. Time axis is presented on the log scale. Data were generated from the S1 of Scenario 2 (with small maximum deviance among the 14 settings in Figure 1) and subject to error $p_0 = 0.003$ and $p_1 = 0.9$. (Sample size N = 100)

Table S3: Region-based p-values for covariates "RA" and "Tcell" on four different regions using the 6 methods under investigation. Because `dmrseq` is designed for WGBS data, for some regions, `dmrseq` reported more than one DMR in the region. Thus, we calculated the `dmrseq`'s pvalue as the minimum over the reported chunks's p-values. The individual p-values for the small chunks within a region are displayed in the last column. The positions of the four regions are given in the left panel of the table.

	CHR	Start	End	nCpG		SOMNiBUS	GlobalTest	BSmooth	SMSC	BiSeq	dmrseq	(chunk p-values from dmrseq)
BANK1	4	102711629	102712832	123	Tcell	5.61E-217	1.48E-15	0.000	0.000	0.000	0.001	(0.001)
					RA	1.10E-08	0.112	0.714	0.700	0.020	0.161	(0.161, 0.318, 0.718)
BLK	8	11350054	11356772	161	Tcell	1.20E-35	0.130	0.158	0.439	0.112	0.001	(0.001, 0.054, 0.251, 0.288)
					RA	7.72E-42	0.924	0.584	0.978	0.529	0.347	(0.347, 0.602, 0.859, 0.979)
HLA_DRB	6	32546614	32557009	61	Tcell	8.89E-250	2.15E-35	0.000	0.000	0.000	3.63E-04	(3.63E-04, 3.63E-04,3.63E-04, 3.63E-04)
					RA	0.029	0.540	0.643	0.966	0.414	0.593	(0.593, 0.618, 0.651, 0.701, 0.714)
PTPN22	1	114353981	114355828	257	Tcell	1.01E-83	5.29E-17	0.000	0.009	0.330	0.001	(0.001, 0.523, 0.54)
					RA	0.045	0.002	0.360	0.136	0.413	0.062	(0.062, 0.693, 0.829, 0.917)

4 SOFTWARE AND DATA

R-package for SOMNiBUS routine: R-package SOMNiBUS contains code to perform the methods described in the article. (GNU zipped tar file) (https://github.com/kaiqiong/SOMNiBUS)

SOMNiBUS Vignette: A user guide of how to use SOMNiBUS package. The vignette also contains the codes for replicating the data example results in this article. (Rmd and HTML files) (https://github.com/kaiqiong/SOMNiBUS/tree/master/vignettes)

Simulation Codes: Codes to replicate the simulation results in the article are deposited in the Github repository https://github.com/kaiqiong/SOMNiBUS_Simu.

References

Balsa, A., Cabezón, A., Orozco, G., Cobo, T., Miranda-Carus, E., López-Nevot, M. Á., Vicario, J. L., et al. (2010). Influence of HLA DRB1 alleles in the susceptibility of rheumatoid arthritis and the regulation of antibodies against citrullinated proteins and rheumatoid factor. *Arthritis research & therapy*, 12(2), R62.

Dempster, A. P., Laird, N. M., and Rubin, D. B. (1977) Maximum likelihood from incomplete data via the EM algorithm. *Journal of the Royal Statistical Society. Series B (Methodological)*, 39(1), 1–22.

Hinks, A., Barton, A., John, S., Bruce, I., Hawkins, C., Griffiths, C. E., et al. (2005) Association between the PTPN22 gene and rheumatoid arthritis and juvenile idiopathic arthritis in a UK population: further support that PTPN22 is an autoimmunity gene. *Arthritis & Rheumatism*, 52(6), 1694–1699.

Oakes, D. (1999). Direct calculation of the information matrix via the EM. *Journal of the Royal Statistical Society: Series B (Statistical Methodology)*, 61(2), 479–482.

Orozco, G., Abelson, A.K., GonzalezGay, M.A., Balsa, A., PascualSalcedo, D., (2009). Study of functional variants of the BANK1 gene in rheumatoid arthritis *Arthritis & Rheumatism: Official Journal of the American College of Rheumatology*, 60(2), 372–379.

Wood, S. N. (2011). Fast stable restricted maximum likelihood and marginal likelihood estimation of semiparametric generalized linear models. *Journal of the Royal Statistical Society: Series B (Statistical Methodology)*, 73(1), 3–36.

Zhang, H., Wang, L., Huang, Y., Zhuang, C., Zhao, G., Liu, R., et al. (2012) Influence of BLK polymorphisms on the risk of rheumatoid arthritis. *Molecular biology reports*, 39(11), 9965–9970.
